# Assessing the Impact of Cyclosporin A on Lentiviral Transduction and Preservation of Human Hematopoietic Stem Cells in Clinically Relevant *Ex Vivo* Gene Therapy Settings

**DOI:** 10.1089/hum.2019.016

**Published:** 2019-09-17

**Authors:** Carolina Petrillo, Andrea Calabria, Francesco Piras, Alessia Capotondo, Giulio Spinozzi, Ivan Cuccovillo, Fabrizio Benedicenti, Luigi Naldini, Eugenio Montini, Alessandra Biffi, Bernhard Gentner, Anna Kajaste-Rudnitski

**Affiliations:** ^1^ San Raffaele Telethon Institute for Gene Therapy (SR-TIGET), IRCCS Ospedale San Raffaele, Milan, Italy; ^2^ Vita-Salute San Raffaele University, School of Medicine, Milan, Italy; ^3^ Gene Therapy Program, Dana-Farber/Boston Children's Cancer and Blood Disorders Center, Boston, Massachusetts; ^4^ Program for Gene Therapy in Rare Diseases, Department of Medicine, Boston Children's Hospital, Boston, Massachusetts.

**Keywords:** lentiviral transduction, human hematopoietic stem cells, transduction enhancers, engraftment and stemness

## Abstract

Improving hematopoietic stem and progenitor cell (HSPC) permissiveness to lentiviral vector (LV) transduction without compromising their biological properties remains critical for broad-range implementation of gene therapy as a treatment option for several inherited diseases. This study demonstrates that the use of one-hit *ex vivo* LV transduction protocols based on either cyclosporin A (CsA) or rapamycin enable as efficient gene transfer as the current two-hit clinical standard into bone marrow–derived CD34^+^ cells while better preserving their engraftment capacity *in vivo*. CsA was additive with another enhancer of transduction, prostaglandin E2, suggesting that tailored enhancer combinations may be applied to overcome multiple blocks to transduction simultaneously in HSPC. Interestingly, besides enhancing LV transduction, CsA also significantly reduced HSPC proliferation, preserving the quiescent G_0_ fraction and the more primitive multipotent progenitors, thereby yielding the highest engraftment levels *in vivo*. Importantly, no alterations in the vector integration profiles could be detected between CsA and control transduced HSPC. Overall, the present findings contribute to the development of more efficient and sustainable LV gene therapy protocols, underscoring the benefits of scaling down required vector doses, as well as shortening the HSPC *ex vivo* culture time.

## Introduction

Recent clinical trials performed in patients affected by primary immunodeficiencies, hemoglobinopathies, and metabolic disorders have shown high levels of gene transfer into hematopoietic stem and progenitor cells (HSPC) that were stably maintained in multiple hematopoietic lineages over a number of years.^1–5^ Nevertheless, early-phase neutropenia after transplantation remains a major cause of treatment-related morbidity in hematopoietic stem cell (HSC) gene therapy and cannot be overcome by increasing the cell dose.^4^ *Ex vivo* culture of HSPC, which typically takes >60 h,^1,2^ may contribute, as increasing evidence shows that cultured HSPC progressively lose engraftment potential through cell-cycle progression and loss of adhesion molecules, thus impairing their homing into the niche and driving lineage commitment and differentiation.^6–9^ Furthermore, it has been shown that the high vector doses currently required for clinically efficacious gene transfer may also *per se* impact *ex vivo* HSPC recovery and their *in vivo* engraftment kinetics due to vector-mediated triggering of the p53 signaling cascade.^10^

On these premises, increasing lentiviral vector (LV) transduction efficiencies would ultimately allow not only the amount of vector required for clinically relevant gene transfer to be decreased, but also the *ex vivo* culture time to be shortened, as well as preserving the biological properties of HSPC, critical for safe and efficient therapeutic outcomes. In this regard, a number of immunomodulatory compounds, including rapamycin (Rapa), cyclosporin A (CsA), and more recently cyclosporin H (CsH), have been identified as capable of significantly increasing LV transduction in both human and murine HSPC.^11–13^ This study assessed the efficacy of the improved CsA- and Rapa-based shorter transduction protocols in clinically relevant settings using bone marrow (BM)-derived CD34^+^ cells and clinical-grade vectors, as well as providing insight regarding the effects of CsA on HSPC engraftment in this context.

## Methods

### Vectors and cells

Third-generation LV stocks were prepared, concentrated, and titered, as previously described.^14,15^ Briefly, self-inactivating (SIN) LV vectors were produced using the transfer vector pCCLsin.cPPT.hPGK.eGFP.Wpre, the packaging plasmid pMDLg/pRRE, Rev-expressing pCMV-Rev, and the vesicular stomatitis virus glycoprotein (VSV-g) envelop-encoding pMD2.VSV-G plasmids. Clinical-grade LVs encoding for the alpha-L-iduronidase *(IDUA)* or the arylsulfatase A *(ARSA)* were produced by MolMed (Milan, Italy) using a large-scale validated process, as previously reported.^2^

The human embryonic kidney 293T cells (HEK293T) used for vector production were maintained in Iscove's modified Dulbecco's medium (IMDM; Sigma–Aldrich, St. Louis, MO) supplemented with 10% fetal bovine serum (FBS; Gibco), penicillin (100 IU/mL), streptomycin (100 μg/mL), and 2% glutamine.

Human CD34^+^ HSPC were isolated through positive magnetic bead selection according to the manufacturer's instructions (Miltenyi Biotec, Bergisch Gladbach, Germany) from umbilical cord blood (CB) collected upon informed consent from healthy volunteers according to the Institutional Ethical Committee approved protocol (TIGET01). Otherwise, CB, BM, or granulocyte colony-stimulating factor (G-CSF) mobilized peripheral blood (mPB) CD34^+^ cells were directly purchased from Lonza (Basel, Switzerland) or HemaCare (Los Angeles, CA). All cells were maintained in a 5% CO_2_ humidified atmosphere at 37°C.

### Transduction

Human CB-derived HSPC were cultured in serum-free StemSpan medium (StemCell Technologies, Vancouver, Canada) supplemented with penicillin (100 IU/mL), streptomycin (100 μg/mL), 100 ng/mL recombinant human stem cell factor (rhSCF), 20 ng/mL recombinant human thrombopoietin (rhTPO), 100 ng/mL recombinant human Flt3 ligand (rhFlt3), and 20 ng/mL recombinant human interleukin-6 (rhIL-6; all from PeproTech, Rocky Hill, NJ) 16–24 h prior to transduction. HSPC were then transduced at a concentration of 1 × 10^6^ cells/mL with VSV-G-pseudotyped SINLV for 16 h at the indicated multiplicity of infection (MOI) in the same medium. BM and G-CSF mPB-derived CD34^+^ cells were placed in culture on retronectin-coated non-tissue culture-treated wells (T100A; Takara Bio, Inc., Kasatsu, Japan) in CellGro medium (CellGenixm Freiburg, Germany) containing a cocktail of cytokines: 60 ng/ml IL-3, 100 ng/mL TPO, 300 ng/mL SCF, and 300 ng/mL FLT-3L (all from Cell Peprotech) for 22–24 h. Cells were then transduced with the indicated dose of vectors for 14–15 h in the same cytokine-containing medium. After transduction with a single-hit reporter LV, cells were washed and maintained in serum-free medium supplemented with cytokines as above until determination of the different subpopulation composition 16 or 72 h later, as well as the percentage of LV-positive cells after 5–7 days by fluorescence-activated cell sorting (FACS), after which they were maintained in IMDM supplemented with 10% FBS, 25 ng/mL rhSCF, 5 ng/mL rhIL6 or rhIL3, 25 ng/mL rhFlt3, and 5 ng/mL rhTPO for an additional 7 days before analysis of vector copy numbers (VCN). In the protocol that foresees two rounds of transduction after the first hit, cells were washed for 10 h in CellGro SCGM medium supplemented with cytokines and underwent a second hit of transduction in the same conditions, as reported previously.^2^ At the end of transduction, cells were counted and collected for clonogenic assays, flow cytometry, and *in vivo* studies. The remaining cells were plated in IMDM 10% FBS with cytokines (60 ng/mL IL-3, 60 ng/mL IL-6, and 300 ng/mL SCF) and cultured for a total of 14 days. Thereafter, cells were collected for molecular and biochemical studies.

### Compounds

CsA and Rapa (both from Sigma–Aldrich) were re-suspended and stored following the manufacturer's instructions. They were added to the transduction medium at 8–10 μM for CsA or 10 μg/mL for Rapa and washed out with the vector 16–20 h later. Where described, 16-16 dimethyl prostaglandin E2 (PGE2; dinoprostone; Yonsung Fine Chemicals Co. Ltd., Hwaseong, South Korea) was added at the final concentration of 10 μM 2 h before LV transduction.

### Colony-forming cell assay and NSG mice

Colony-forming cell (CFC) assays were performed by plating 8 × 10^2^ human HSPC transduced in the presence of the different compounds in a methylcellulose-based medium (Methocult GF4434; StemCell Technologies). Fifteen days later, colonies were scored by light microscopy for colony numbers and morphology as erythroid or myeloid and were collected both as a pool and picked as a single colonies and lysed for molecular analysis to evaluate transduction efficiencies with clinical-grade LVs.

Female NOD-SCID-IL2Rg^−/–^ (NSG) mice were purchased from Jackson Laboratory. All animal procedures were performed according to protocols approved by the Animal Care and Use Committee of the Ospedale San Raffaele (IACUC 782) and communicated to the Ministry of Health and local authorities according to the Italian law. Human BM-derived CD34^+^ cells were pre-stimulated and transduced as described before with clinical-grade *IDUA*-LV (MolMed SpA, Milan, Italy) at a MOI of 100 or with lab-grade SINLV-GFP at a MOI of 10 in the presence or absence of dimethyl sulfoxide (DMSO)/CsA/Rapa as indicated. After transduction, 2–5 × 10^5^ cells were infused into the tail vein of sublethally irradiated 8- to 10-week-old NSG mice (radiation dose: 200 cGy for mice weighing 18–25 g and 220 cGy for mice weighing >25 g). Transduced cells were also cultured *in vitro* for 14 days for further analysis. *In vitro* cultured cells and BM or brain-repopulating cells isolated from transplanted mice at the time of sacrifice were then used to quantify the VCN by quantitative polymerase chain reaction (PCR).

### Genomic DNA extraction and Droplet Digital PCR

DNA from human CD34^+^ liquid culture, hematopoietic colony pool samples, and murine tissues was extracted using a Maxwell 16 instrument (Promega, Madison, WI) or Blood & Cell Culture DNA micro kit (Qiagen, Valencia, CA). DNA from single colonies was lysed in Monini buffer, as previously described.^16^ VCN of the integrated lentiviral vectors were quantified by quantitative droplet ddPCR using the following primers against the primer binding site region of LVs: HIV sense 5′-TACTGACGCTCTCGCACC-3′; HIV antisense: 5′-TCTCGACGCAGGACTCG-3′; and probe FAM 5′-ATCTCTCTCCTTCTAGCCTC-3′. VCN were normalized to genomic DNA content, which was assessed using the human *TERT* gene, as previously described.^11^ VCN analysis by Droplet Digital PCR (ddPCR) involved quantification of target and reference loci through the use of duplex target and reference assays. In QuantaSoft™ software, the copy number was determined by calculating the ratio of the target molecule concentration to the reference molecule concentration multiplied by the number of copies of reference species in the genome (usually two). Transduction efficiencies were evaluated by ddPCR on individual colonies from CFC assay performed on the HSCs transduced with clinical-grade LVs and expressed as a percentage of LV^+^ colonies on the total tested, as previously reported.^2^ The PCR reaction for ddPCR is reported in Supplementary Table S1.

### Integration site retrieval and sequencing

The genomic DNA portions flanking the vector integration sites were retrieved by linear amplification–mediated (LAM) PCR on ∼300 ng of genomic DNA fragmented with MluCI, HpyCHIV4, and AciI restriction enzymes, as previously described,^17^ and ligated to a restriction site–complementary linker cassette containing an eight nucleotide sequence barcode used for sample identification, a random 12 nucleotide sequence needed for improved cluster recognition, and all the sequences required for the Read 2 Illumina paired-end sequencing. LAM-PCR products were purified by AmpureXP beads and quantified with a Qubit™ Fluorometer (Thermo Fisher Scientific, Pittsburgh, PA). Forty nanograms of PCR product was re-amplified with Fusion-LTR (AATGATACGGCGACCACCGAGATCTACACTCTTTCCCTACACGACGCTCTTCCGATCTNNNNNNNNNNNNXXXXXXXXACCCTTTTAGTCAGTGTGGA) and Fusion-LC (CAAGCAGAAGACGGCATACGAGATGTGACTGGAGTTCAGACGTGTGCTCTTCCGATCTNNNNNNNNNNNNXXXXXXXXgatctgaattcagtggcacag). LAM-PCR amplicons were separated on a Shimadzu MultiNA Microchip Electrophoresis System to evaluate the presence of PCR amplification products. Moreover, an additional PCR method developed by Firouzi *et al*. was adopted.^18^ Briefly, the genomic DNA from transduced cells was fragmented by adaptive focused acoustic sonication using a Covaris E220 Ultrasonicator (Covaris, Inc., Woburn, MA). The fragmented DNA was then subjected to end repair and 3′ adenylation, ligated to the DNA blunted-end linker cassette using the NEBNext^®^ Ultra™ DNA Library Prep Kit for Illumina^®^ following the manufacturer's instructions (New England Biolabs, Ipswich, MA). The ligation products were purified with AmpureXP beads and then divided into three parts (300 ng each) in order to obtain technical triplicates and re-amplified using the oligonucleotides Fusion-LTR and Fusion-LC described above. The last exponential PCR was performed using Qiagen TAQ DNA polymerase under the following conditions: 95°C for 2 min, and 95°C for 45 s, 58°C for 45 s, and 72°C for 1 min for 12 cycles, followed by a further 5 min of incubation at 72°C. Barcoded PCR products were quantified by fluorometric quantification and assembled into libraries in equimolar ratios, avoiding repetition of identical pairs.

### Integration site mapping and bioinformatics analysis

The vector integration sites were mapped on the human genome (version hg19) using VISPA2, a bioinformatics pipeline designed to generate a the list of insertion sites and the nearest annotated RefSeq gene.^19^ Filtering and normalization procedures to remove potential PCR contaminations with other independent studies and clonal abundance measurements were performed, as described previously.^1,2^ Common insertion sites (CIS) were identified by the kernel convolution framework method developed by De Ridder *et al*.^20^ Enrichment analysis for ontological classes among the vector targeted genes was performed by the Genomic Regions Enrichment of Annotations Tool.^21^

### Flow cytometry

All cytometric analyses were performed using the FACSCanto instrument and LSRFortessa instruments (BD Biosciences, Franklin Lakes, NJ) and analyzed with FACS Express (De Novo Software, Glendale, CA). For antibodies, see Supplementary Table S2.

#### Transduced cells

Green fluorescent protein (GFP) expression in transduced cells was measured 5–7 days post transduction. To measure HSPC subpopulation composition, cells were harvested 16 or 72 h post transduction, incubated with anti-human receptor blocking antibodies for 15 min at 4°C, and then stained for 20 min at 4°C with anti-human CD34, CD38, CD45RA, and CD90 or with anti-human CD34, CD133, and CD90 antibodies. To exclude dead cells from the analysis, 10 ng/mL 7-aminoactinomycin D was added.

#### Analysis of NSG mice

For each mouse, cells obtained from peripheral blood or BM were first incubated with anti-human FcγIII/II receptor (Cd16/Cd32) blocking antibodies for 15 min at 4°C and then incubated in the presence of monoclonal antibodies (Supplementary Table S2) for 20 min at 4°C. Where necessary, erythrocytes were removed by lyses with the TQ-Prep workstation (Beckman Coulter, Brea, CA) in the presence of an equal volume of FBS (100 μL) to protect white blood cells. Cells were finally washed and re-suspended in PBS containing 2% FBS. For brain analysis, mice were euthanized under deep anesthesia followed by extensive intracardiac perfusion with cold PBS for 15 min after clamping the femur. Organs were then collected and differentially processed, as previously reported.^22^ For the analysis of hCD34^+^-derived cell engraftment in the brain, an enrichment for myeloid cells was performed using a 30% Percoll gradient^23^ after enzymatic (19 mg papain, 10 mg cystein, 2.5 mg DNAse) digestion of the brain tissue.

#### Ki67 and Hoechst flow cytometry

Cells were washed and fixed using BD Cytofix buffer (cat. #554655), washed and permeabilized with BD Perm 2 (cat. #347692), washed and stained with PE-conjugated Ki67 antibody (BD Biosciences), and finally re-suspended in BD Cytofix buffer with Hoechst at 1 μg/mL. The cells were then analyzed on a BD LSRII machine with UV laser.

#### Cell proliferation assay

Cells were stained with Cell Proliferation Dye eFluor^®^ 670 (Affimetrix; eBioscience, San Diego, CA) after 24 h of cytokine pre-stimulation and before cell transduction. This fluorescent dye binds to any cellular protein containing primary amines, and as cells divide, the dye is distributed equally between daughter cells that can be measured as successive halving of the fluorescence intensity of the dye. At different time points after transduction, cells were harvested and analyzed with flow cytometry. Cell Proliferation Dye eFluor^®^ 670 has a peak excitation of 647 nm and can be excited by the red (633 nm) laser line. It has a peak emission of 670 nm and can be detected with a 660/20 band pass filter (equivalent to APC, Alexa Fluor^®^ 647, or eFluor^®^ 660).

#### Reactive oxygen species quantification

Cells were stained with CM-H2DCFDA (Thermo Fisher Scientific), which passively diffuses into cells, where its acetate groups are cleaved by intracellular esterases and its thiol-reactive chloromethyl group reacts with intracellular glutathione and other thiols. Subsequent oxidation yields a fluorescent adduct that is trapped inside the cell and monitored using a flow cytometer. N-acetyl-L-cysteine and hydrogen peroxide (both from Sigma–Aldrich) were added with the fluorescent probe at a concentration of 1 and 10 mM, respectively.

### Statistical analysis

In all studies, values are expressed as the mean ± standard deviation. Replicates internal to each individual experiment were first averaged and then combined with the other independent experiments. For analysis of two groups, an unpaired nonparametric Mann–Whitney test was used. For experiments containing more than two groups, a nonparametric one-way analysis of variance was used. More specifically, the unpaired nonparametric Kruskal–Wallis test with Dunn's multiple comparison was used to compare unmatched observations (*i.e.*, individual animals/CFU) from multiple treatments groups to the control group (DMSO or two-hit, as indicated). Instead, for matched observations (*i.e.*, different treatments performed in parallel on cells from a single donor), the nonparametric paired Friedman test with Dunn's multiple comparison was used. Differences were considered statistically significant at *p* < 0.05.

## Results

### Impact of one-hit CsA and Rapa-based protocols on gene transfer efficiency and HSPC engraftment over the current standard

**Figure 1.**
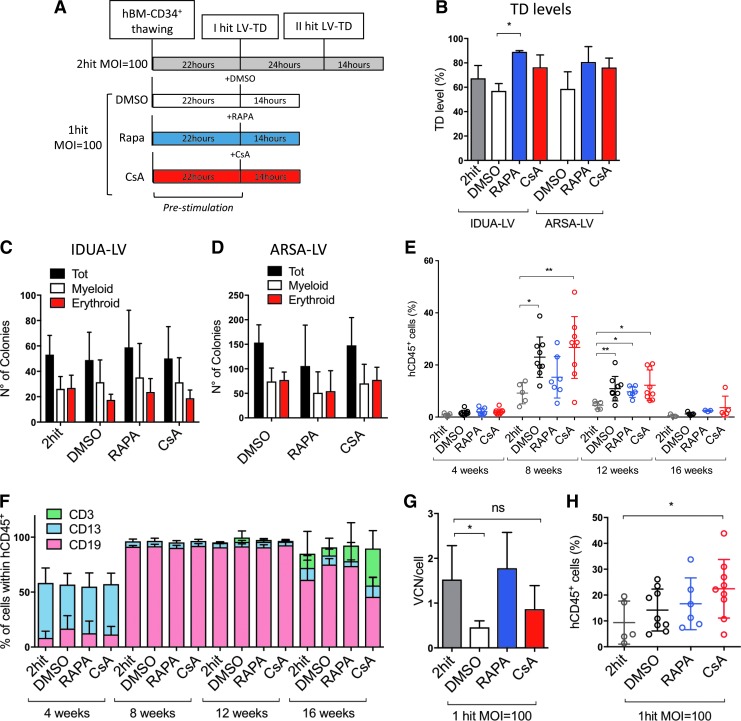
Impact of one-hit cyclosporin A (CsA) and rapamycin (Rapa)-based protocols on gene transfer efficiency and hematopoietic stem and progenitor cell (HSPC) engraftment over the current standard. **(A)** Scheme of the experimental approach comparing the different transduction protocols using two clinical-grade lentiviral vectors (LVs) and bone marrow (BM)-derived CD34^+^ cells. **(B)** The percentage of transduction (*M* ± standard deviation [*SD*], *n* = 3, Friedman with Dunn's multiple comparisons test vs. dimethyl sulfoxide [DMSO]; *p ≤ 0.05), as well as **(C** and **D)** the number of colony-forming units counted 15 days after plating are shown for cells transduced with alpha-L-iduronidase *(IDUA)* or arylsulfatase A *(ARSA)*-encoding LV. **(E** and **F)** Peripheral blood analyses in NSG mice at different times post transplant. **(E)** Engraftment levels evaluated as percent of human CD45^+^ cells over the total of blood mononuclear cells (*y*-axis) in mice from different treatment groups (indicated on the *x*-axis; *M* ± *SD*, *n* ≥ 5, Kruskal–Wallis with Dunn's multiple comparisons vs. two-hit; *p ≤ 0.05, **p ≤ 0.01). **(F)** Percentages of human B, T, and myeloid cell linages (hCD19^+^, hCD3^+^, and hCD13^+^, respectively) within human CD45^+^ cells were shown over time. **(G)** Vector copy number (VCN) and **(H)** engraftment levels of human CD45^+^ cells were shown in the BM of mice at 22 weeks post transplant (*M* ± *SD*, *n* ≥ 5, Kruskal–Wallis with Dunn's multiple comparisons vs. two-hit; ns, not significant; *p ≤ 0.05).

To investigate the potential of CsA and Rapa to enhance LV transduction in a clinically relevant setting, human BM-derived CD34^+^ cells were transduced, in the presence or absence of the compounds, with a single dose of clinical-grade LV at a MOI of 100 encoding either the *IDUA* or the *ARSA* transgene, developed for the treatment of type I mucopolysaccharidosis (MPS-1) and metachromatic leukodystrophy (MLD), respectively.^2,24^ As a reference for the *IDUA*-LV protocol, cells were also transduced with the current standard transduction protocol, which consists of two rounds of transduction at a MOI of 100 of vector (Fig 1A). CsA and Rapa were able to increase the transduction efficiency of both clinical-grade LV compared to the one-hit control without drugs (Fig. 1B). Of note, Rapa seemed to yield an increased proportion of colonies harboring very high VCN compared to the other conditions (Supplementary Fig. 1A). CsA, on the other hand, seemed to increase the overall percentage of vector-marked cells rather than the VCN in a small fraction of the population (Fig. 1B and Supplementary Fig. 1A). No alterations in colony-forming capacity were observed (Fig. 1C and D). However, some degree of toxicity was noticed in CsA-exposed mPB-derived HSPC confirmed by higher *ex vivo* apoptosis and lower colony output (Supplementary Fig. 1B and C) compared to what was observed for HSPC from other cell sources here and in previous studies.^11^

Next, the *IDUA*-LV transduced CD34^+^ cells were transplanted into NSG mice, and the extent of vector marking in human hematopoietic cells was assessed in the long-term repopulated mice. Of note, no significant differences in the numbers of HSPC counted at time of transplantation were detected among the different treatment groups (Supplementary Fig. 1D), indicating no overt toxicity of the tested transduction protocols. FACS analysis of the peripheral blood of NSG mice transplanted with one-hit transduced HSPC showed better engraftment of human CD45^+^ cells compared to the standard protocol up to 16 weeks post transplant, in particular with CsA (Fig. 1E). Moreover, no significant alterations in the percentages of human B (hCD19^+^), T (hCD3^+^), and myeloid (hCD13^+^) lineage outputs were observed (Fig. 1F). Both CsA and Rapa enabled improved transduction of long-term repopulating HSPC *in vivo* as VCN/human genome comparable or not significantly different to the two-hit standard protocol were reached with half of the vector dose in the BM of the mice 22 weeks post transplantation (Fig. 1G). Importantly, significantly improved HSPC engraftment was also maintained in the BM of the mice for CsA-exposed cells (Fig. 1H). No significant differences were observed in the different lineage outputs compared to controls in the BM of the mice long term (Supplementary Fig. 1E).

### Vector integration site analysis in transduced HSPC shows a positive safety profile of CsA treatment *in vitro* and *in vivo*

**Figure 2.**
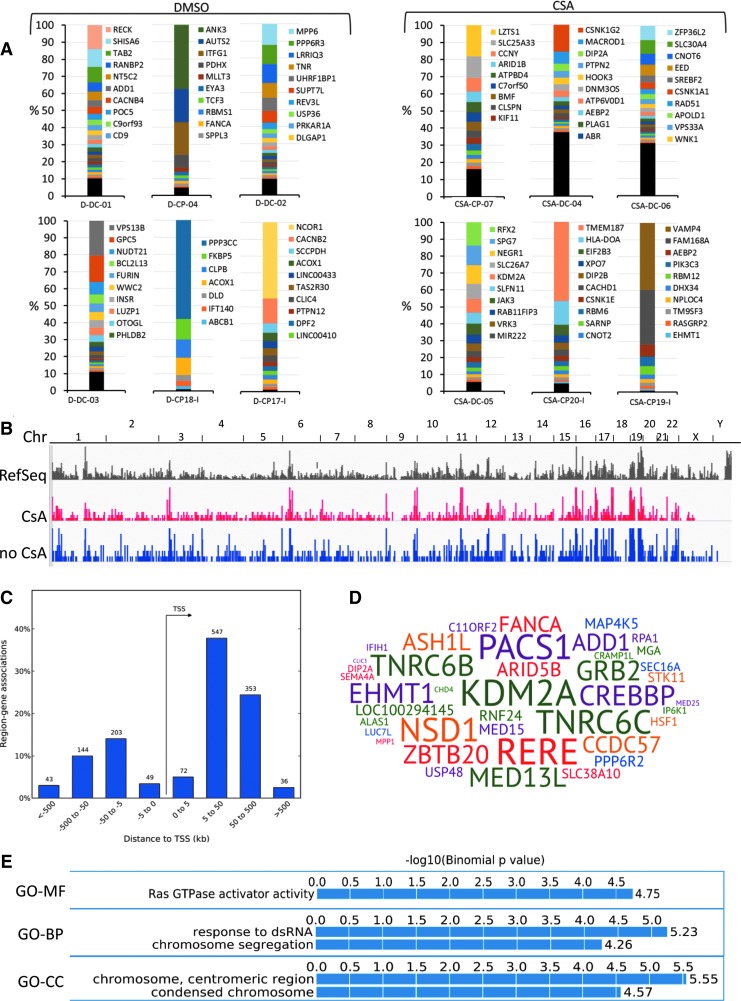
Integration site analysis in HSPC transduced in the presence or absence of CsA. **(A)** Bar-plot representation of clonality and clonal abundance in BM cells harvested from NSG mice 17 weeks after transplant with human CD34^+^ cells transduced in the presence or absence of CsA (indicated as CsA and DMSO, respectively). Each color in the abundance bars represents the relative percent of integration sites with an abundance >1%; most abundant integrations are on *top*. Integration sites with an abundance <1% were pooled, and are represented in *black* at the bottom of each bar. The genes targeted by the 10 most abundant integration sites are indicated on the *left* of each bar (most abundant ranked from top to bottom). Mice D-CP18-I, D-CP17-I, CSA-CP20-I, and CSA-CP-19-I were transplanted with HSPC transduced with the IDUA-LV, while the remaining mice were transplanted with SINLV-green fluorescent protein *(GFP)*. **(B)** Frequency distribution in chromosomes (indicated on *top*) of Ref Seq genes (in *gray*) and SIN-LV*-GFP* insertions in *in vitro* cultured HSPC after transduction in the presence of CsA (in *red*) or HSPCs in *in vitro* cultured HSPC transduced with the *ARSA*-expressing LV from a patient from the metachromatic leukodystrophy clinical trial (in *blue*; from Biffi *et al*.^2^). **(C)** Frequency distribution (*y*-axis, in %) of LV integration sites around gene transcription start sites (*x*-axis, in Kb). Above each bar, the number of integration sites landing in the specific interval is indicated. **(D)** Word cloud representation of the genes significantly over-targeted by common insertion site statistics in *in vitro* cultured HSPC after transduction with SINLV*-GFP* in the presence of CsA. The size of each gene symbol is proportional to the number of targeting LV insertions. **(E)** Enrichment analysis for ontological classes among the vector targeted genes performed by the Genomic Regions Enrichment of Annotations Tool (GREAT). GO, gene ontology; MF, molecular function; BP, biological process; CC, cellular component.

To characterize the safety and efficacy of CsA treatment further, the study investigated the LV genomic integration profile in blood/BM cells harvested from NSG mice after 17 weeks from transplantation with human BM-derived HSPCs *ex vivo* transduced in the presence or absence of CsA with a lab-grade SINLV*-GFP in vitro* (a MOI of 10) or GLP-grade *IDUA*-LV (a MOI of 100; Supplementary Table S3). The genomic DNA portions flanking the LV insertion sites were retrieved by state-of-the-art LAM (17) and a sonication-based linker-mediated PCR method,^18^ sequenced by Illumina technology, and mapped on the human genome (version hg19) by a dedicated bioinformatics pipeline.^19^ Overall, the study mapped >2,700 integration sites from *in vitro* cultured HSPCs transduced with or without CsA and BM cells of mice transplanted with HSPCs transduced in the presence (*n* = 6) or absence of CsA (*n* = 6; Fig. 2A). The number of insertions retrieved in CsA treatment groups, after normalization for the amount of total genomes analyzed, was significantly higher in both *in vitro* and *in vivo* data sets (*in vitro*: 1,446 CsA integration sites from 500 ng of DNA vs. 291 DMSO integration sites from 167 ng of DNA; *in vivo*: 671 CsA integration sites from 1,667 ng of DNA in vs. 354 DMSO integration sites from 1,200 ng of DNA; *p* < 0.0001). Although most of the mice at 17 weeks after transplant showed oligoclonal hematopoiesis, mice in the CsA treatment groups had an increased clonality (Fig. 2A). The distribution of the LV insertions in CsA-treated HSPCs, at the genome-wide level, showed a tendency to integrate in gene-dense regions, with a strong clustering of insertions in megabase-wide intervals in specific chromosomal regions (Fig. 2B). On a smaller scale, LV insertions in the CsA treatment groups showed a strong tendency to integrate within genes without biases for promoter regions (Fig. 2C). The analysis of the gene-targeting frequency by CIS statistics (Methods) showed that among the most targeted genes by LV insertions in the CsA treatment group were *KDM2A*, *RERE*, *TNRC6C*, *TNRC6C*, *PACS1*, and *NSD1* (Fig. 2D). Finally, enrichment analysis for ontological terms of the genes targeted by LV integration in the CsA treatment group did not show any bias for cancer-related genes (Fig. 2E).

### Combinatorial potential of CsA and Rapa with PGE2 to enhance HSPC transduction

**Figure 3.**
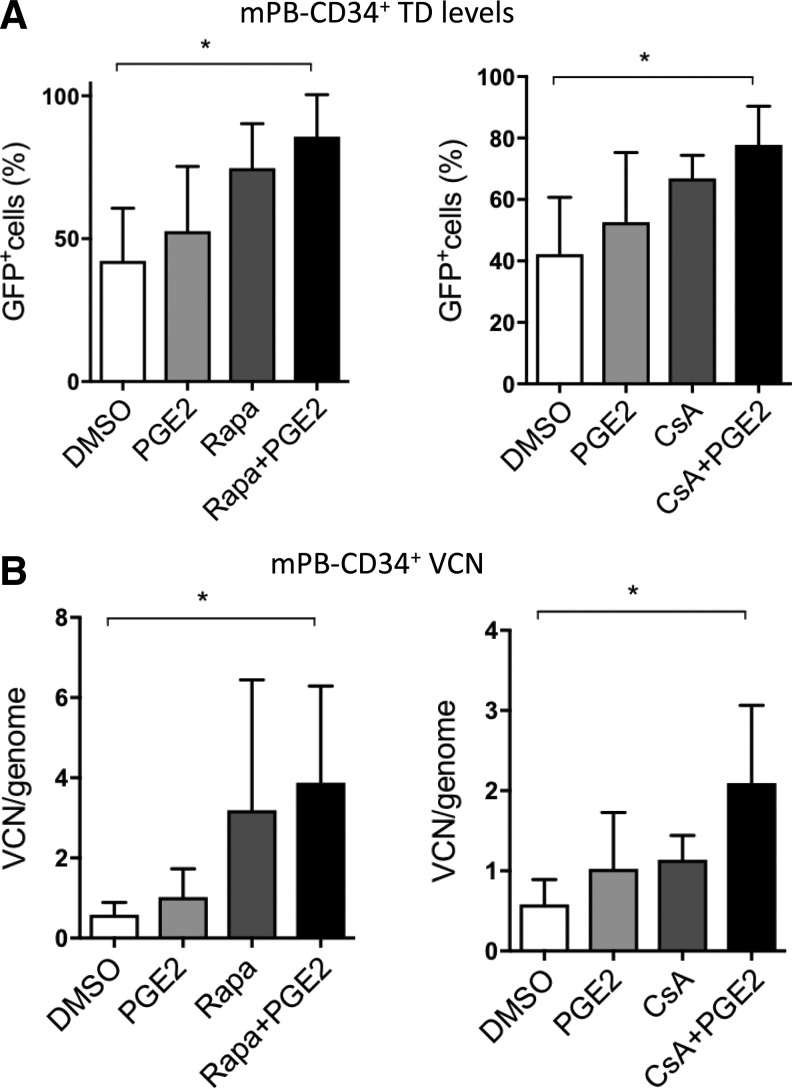
CsA and prostaglandin E2 have an additive effect on LV transduction. Mobilized peripheral blood (mPB)-CD34^+^ cells were transduced with a SIN-LV PGK*-GFP* at a multiplicity of infection (MOI) of 10 in the presence or absence of the compounds or their combination. **(A)** The percentage of GFP^+^ cells was evaluated by fluorescence-activated cell sorting (FACS) 5 days after transduction. **(B)** VCN were assessed 14 days after transduction. Data represent the mean ± *SD*, *n* = 3, Friedman with Dunn's multiple comparisons test vs. DMSO; *p ≤ 0.05.

Another small molecule, PGE2, has been recently shown to enhance VSV-G-pseudotyped LV transduction of human HSPC.^9,25^ Both Rapa and PGE2 likely act at the level of vector entry into HSPC, although their precise mechanisms of actions remain unclear.^9,11,13,25^ To test the combinatorial potential of the compounds with PGE2 to enhance LV transduction, mPB-derived CD34^+^ cells were transduced with SINLV*-GFP* at a MOI of 10 in the presence or absence of the compounds alone or in combination. Interestingly, PGE2 was additive with both Rapa and CsA in terms of percentage of vector-positive cells (Fig. 3A), while the additive effects were more pronounced for CsA in terms of VCN (Fig. 3B).^11^ Taken together, these results suggest that specific compound combinations may be tailored to overcome multiple blocks to transduction simultaneously in HSPC.

### CsA-mediated improved transduction is maintained in the HSPC-derived brain progeny

**Figure 4.**
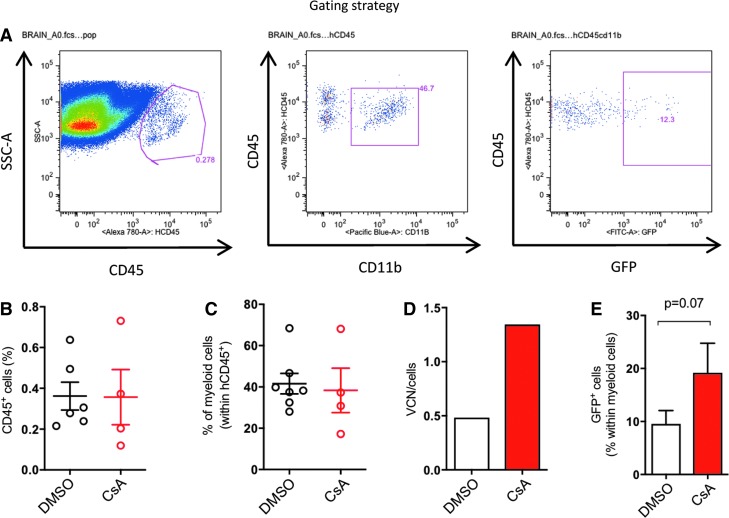
CsA also improves transduction in brain-repopulating myeloid progenitors. BM-CD34^+^ cells were transduced with SINLV PGK*-GFP* at a MOI of 10 with or without CsA and were then transplanted into NSG mice. **(A)** Brain analysis of the mice was performed at sacrifice 17 weeks post transplant, as reported in the scheme. **(B)** Percentages of human CD45^+^ over the total cells were evaluated. **(C)** Percentages of human myeloid cell lineages (hCD11b^+^) within human CD45^+^ cells are shown. **(D)** VCN from colony-forming units 15 days after plating are reported. **(E)** Transduction levels were evaluated as percentage of GFP^+^ cells with FACS. Data represent the mean ± *SD*, *n* ≥ 4 mice per group, Mann–Whitney test.

A key feature of *ex vivo* gene therapy approaches to treat lysosomal storage disorders (LSD) such as MLD and MPS-1 is the capacity of transduced HSPC to contribute to brain myeloid populations upon engraftment, thus allowing cross-correction of the tissue-specific defects through supraphysiological expression of the transgene by the gene-modified progeny.^2,26^ With this in mind, the study investigated the impact of CsA on the levels of vector marked HSPC–derived cells in the brain. Briefly, BM-derived CD34^+^ cells were transduced with a SINLV*-GFP* at a MOI of 10 in the presence or absence of CsA and injected into sublethally irradiated NSG mice. Mice were sacrificed at 17 weeks for analysis of the brain (Fig. 4A). Both treatment groups showed comparable engraftment levels in the brain (Fig. 4B), and approximately 40% of the human graft in the brain stained for the myeloid marker CD11b in both treatment groups (Fig. 4C). Importantly, higher vector marking was observed for the CsA-treated group and was also maintained in the brain progeny of the transplanted HSPC (Fig. 4D and E), further highlighting the benefits CsA could potentially provide in the context of *ex vivo* gene therapies for LSD.

### CsA reduces HSPC proliferation and preserves quiescence in culture

**Figure 5.**
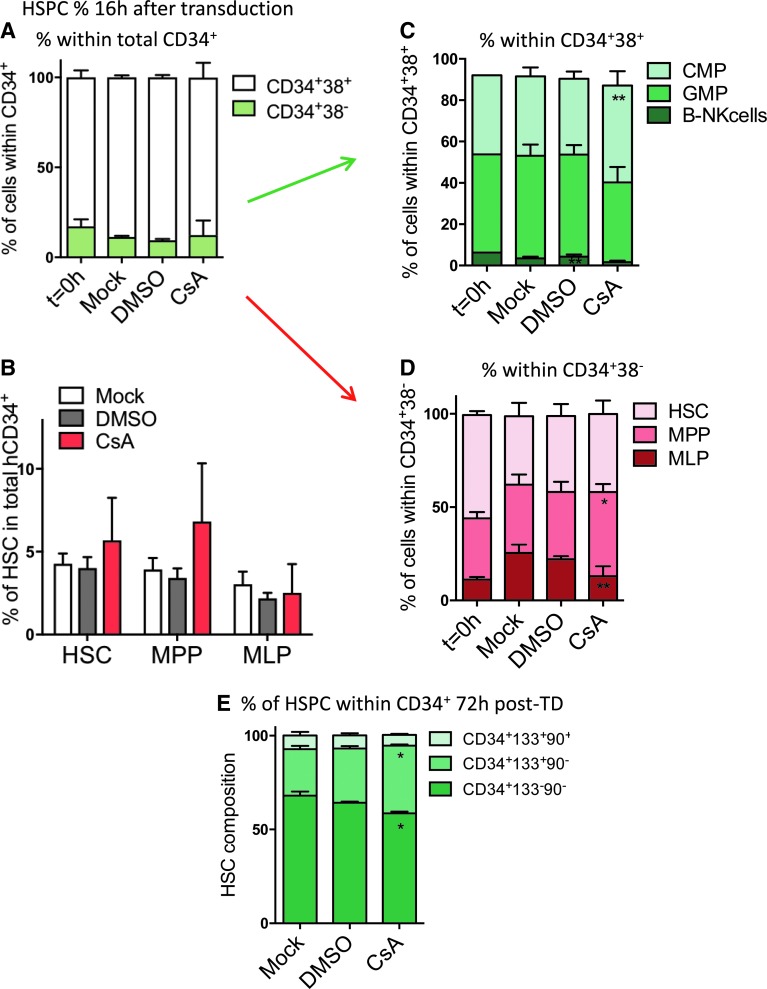
CsA preserves primitive HSC *ex vivo.* Human BM-derived CD34^+^ cells were left untransduced (mock) or were transduced with SINLV*-GFP* at a MOI of 10 in the presence of CsA or the carrier control DMSO. The subpopulation composition of BM-derived CD34^+^ HSPC was measured at 0 or 16 h **(A–D)** and 72 h **(E)** post transduction. Data represent the mean ± *SD*, *n* = 4, Friedman with Dunn's multiple comparisons test versus mock; *p ≤ 0.05, **p ≤ 0.01. The *green arrow* indicates that the analysis in **C** has been done on the CD34+CD38+ fraction, represented by the *white* part of the bars in **A**. The *red arrow* indicates that the analysis in **D** regards the DC34+DC38- fraction, represented by the *green* portion of the bars in **A**. HSC, hematopoietic stem cell; MPP, multipotent progenitor; MLP, multipotent lymphoid progenitor; CMP, common myeloid progenitor; GMP, granulocyte-monocyte progenitor.

To investigate the reasons behind higher engraftment of human cells observed for the CsA condition, the impact of CsA on the stem-cell composition *in vitro* was evaluated. Human BM-derived CD34^+^ cells were left untransduced (mock) or were transduced with SINLV*-GFP* at a MOI of 10 in the presence of CsA or the carrier control DMSO. The percentage of the primitive stem and progenitor cells was evaluated by FACS 16 h post transduction (Fig. 5A–D and Supplementary Fig. 2A). CsA exposure *in vitro* tended to preserve the percentage of more primitive stem and multipotent progenitors (Fig. 5B–D) with a significantly higher percentage of common myeloid progenitor (CMP; Fig. 5C) and multipotent progenitors (MPP; Fig. 5D). The impact of CsA on the multipotent CD34^+^CD133^+^CD90^+^ progenitors was maintained up to 72 h post transduction *in vitro* (Fig. 5E and Supplementary Fig. 2B).

**Figure 6.**
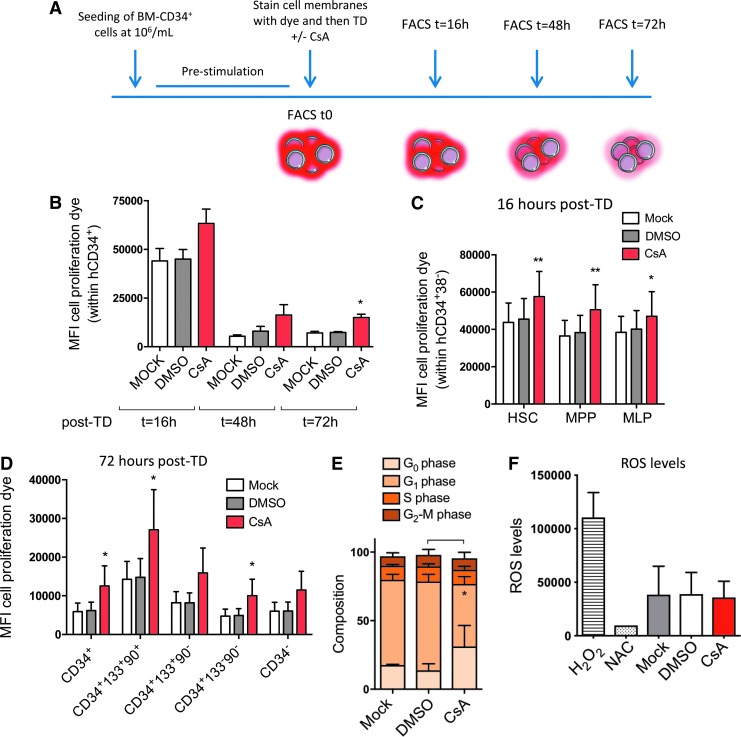
CsA reduces HSPC proliferation and preserves quiescence in culture. Human BM-derived CD34^+^ cells were left untransduced (mock) or were transduced with SINLV*-GFP* at a MOI of 10 in the presence of CsA or the carrier control DMSO. **(A)** BM-derived CD34^+^ cells were stained before LV exposure ± CsA with a red fluorescent dye that can be used to monitor individual cell divisions. The mean fluorescence intensity of the dye was monitored over time by FACS in **(B)** the total CD34^+^ population (*M* ± *SD*, *n* ≥ 3, Kruskal–Wallis with Dunn's multiple comparisons versus mock; *p ≤ 0.05) and within the different subpopulations at **(C)** 16 h and **(D)** 72 h of culture (*M* ± *SD*, *n* = 4, Friedman with Dunn's multiple comparisons test vs. mock; *p ≤ 0.05, **p ≤ 0.01). **(E)** Cell-cycle status (*M* ± *SD*, *n* = 3, Friedman with Dunn's multiple comparisons test vs. mock; *p ≤ 0.05) and **(F)** reactive oxygen species levels of BM-derived HSPC were assessed 48 h post transduction.

To investigate whether slower proliferation rates could explain the preservation of the more primitive cells in the presence of CsA, human BM-derived CD34^+^ cells were stained with a fluorescent dye that can be used to monitor individual cell division. Following this staining, cells were transduced with or without CsA, and the levels of cell proliferation dye within the total and the different HSPC subpopulations were evaluated by FACS at different times post transduction (Fig. 6A). CsA significantly reduced cell proliferation over time in the total CD34^+^ population (Fig. 6B), as well as within all the different subsets, ranging from the more committed progenitors to the most primitive cells (Fig. 6C and D).

As the primitive HSC are characterized by a more quiescent cell-cycle status,^27^ the study investigated whether the CsA-mediated increase in more primitive stem and multipotent progenitors was also associated with a higher fraction of cells in the G_0_ phase of the cell cycle. A FACS-based combinatorial staining strategy was used that allowed cells in G_0_, G_1_, S, and G_2_-M phases of the cell cycle to be distinguished. CsA exposure significantly increased the proportion of cells arrested in the quiescent G_0_ phase of the cycle (Fig. 6E). As oxidative stress plays a critical role in HSPC biology and CsA has been suggested to impact the redox balance in HSPC,^28^ the effects of CsA on reactive oxygen species (ROS) levels in human BM-derived HSPC were also evaluated. In the experimental setting, CsA did not lead to any alterations in ROS levels measured *in vitro* (Fig. 6F). Taken together, the CsA-mediated decrease in HSPC proliferation and maintenance of quiescence could potentially contribute to preserving their stem-cell properties, yielding higher engraftment *in vivo*.

## Discussion

Improving cell recovery as well as rapid and robust engraftment of *ex vivo* manipulated HSPC remains a high-priority goal for the safe and successful clinical deployment of HSC gene therapy. Several efforts to improve LV transduction efficiencies in human HSPC have led to the identification of a number of small molecules capable of enhancing gene transfer into CD34^+^ cells.^9,11–13,25^ The present study demonstrates the efficacy and safety of CsA and Rapa in clinical culture conditions using BM-derived HSPC and clinical-grade LV. In particular, these protocols allowed clinically relevant levels of transduction in long-term repopulating HSC to be achieved with two times less vector and in a significantly shorter time of *ex vivo* culture.

Improved one-hit transduction protocols such as the CsA-based approach have the potential to benefit *ex vivo* HSC gene therapy significantly. Achieving clinically efficacious transduction levels with one single round of transduction while better preserving HSPC *ex vivo* and improving their engraftment *in vivo* would ultimately allow the treatment of more patients with a given vector batch compared to current clinical standards. Robust gene transfer into HSC might also allow the intensity of the conditioning regimen to be reduced and non-genotoxic and stem cell–specific conditioning strategies to be implemented.^29^ Moreover, in the case of LSD, preclinical work in animal models has demonstrated the superior benefit associated with the transplantation of LV-transduced HSPC expressing supra-normal enzyme levels instead of wild-type cells in correcting disease manifestations,^26,30–32^ supported by preliminary data for MLD in recent clinical trials.^2,4^ The superior outcome of HSC gene therapy in animal models is due to greater enzyme delivery and cross-correction of the affected tissues, including the brain.^26,30^ In this setting, the maintenance of increased gene transfer achieved with CsA in the brain progeny of the transplanted HSPC would potentially allow the vector dose to be lowered and the *ex vivo* transduction protocol to be shortened without compromising enzyme delivery to the brain—crucial for clinical benefit.

CsA yielded a 3.4-fold increase in vector copies *in vitro* using mPB-CD34^+^ cells when combined with the early-acting enhancer of transduction PGE2, indicating that there remains a further margin to increase gene transfer efficiency into HSPC with tailored drug combinations. In agreement, preserving beneficial host–vector interactions further potentiates the capacity of cyclosporin to enhance transduction in HSPC, as recently shown for CsH^12^ that differently from CsA does not interfere with the interaction of the vector capsid with the host factor cyclophilin A.^33^ Also, Rapa was additive with PGE2, although to a lesser extent than CsA in terms of gene marking, potentially suggesting that both compounds act on events different from PGE2 during vector entry into target cells. In agreement, both cyclosporin and Rapa have been recently suggested to target the same entry block into HSPC,^12,34^ although their precise mechanisms of action still remain to be elucidated. Of note, some degree of toxicity was noticed in this specific cell source when transducing them in the presence of CsA. A potential explanation for these differences observed between BM and mPB-derived HSPC may reside in the mobilization protocols used to isolate mPB-derived CD34^+^ cells. G-CSF, the most commonly used agent for HSPC mobilization, is induced in response to infection or inflammation.^35^ It is possible that mobilization causes some degree of inflammatory priming of HSPC, rendering them more sensitive to external cues, including vector signaling, and apoptosis. Moreover, the calcineurin-mediated immunosuppressive effects of CsA may also contribute to the observations in mPB-CD34^+^ cells, as the calcineurin-independent cyclosporin CsH did not affect mPB-CD34^+^ cell survival or long-term repopulating capacity *in vivo* in a recent study.^12^ The calcineurin-mediated immunosuppressive effects of CsA together with other cell source-specific effects could also explain the slight albeit insignificant impact CsA had on the CD3^+^ T-cell output *in vivo* using BM-derived HSPC, as such effects were not observed in the context of previous studies using CB–derived HSPC and lab-grade vectors^11^ or CsH and mPB-derived cells.^12^

Both Rapa and PGE2 have been reported not to impact the LV integration site profile in human HSPC.^25^ CsA instead has been shown to impact HIV-1 integration profiles in somatic cell lines due to its effects on the interaction of the viral capsid with the host factor cyclophilin A,^36^ but no reports in the context of HSPC are available to date. The present study evaluated the safety profile of the CsA-based gene transfer protocol in terms of LV integration site distribution. Although more integration sites were systematically retrieved in the presence of CsA *in vitro* and *in vivo*, in agreement with its ability to increase vector copies per cell, no significant impact of CsA treatment on LV integration profiles was detected in the context of HSPC. Moreover, the LV integration profile in CsA-treated HSPC was highly similar to that described in other integration site studies obtained from humanized mouse models and recent gene therapy trials,^1–3,37^ which maintain a positive safety profile after several years of follow-up from transplant, further supporting the safety of CsA-based transduction protocols. This feature, together with the propensity of CsA to enhance transduction through a homogeneous increase in the percentage of vector-marked cells rather than through the generation of few cells harboring very high VCN, support its application in future gene therapy protocols based on BM-derived HSPC.

Besides improving BM-derived HSPC transduction, CsA also tended to ameliorate its engraftment capacity *in vivo*, in particular early post transplantation. This benefit is likely related to its capacity to decrease HSPC proliferation and better preserve the more primitive cells in culture, as both quiescence and shorter *ex vivo* culture have been shown to improve HSPC engraftment in other settings.^6–9^ CsA has also been shown to improve murine Lin^−^ and human CB-derived CD34^+^ cell engraftment through its capacity to dampen cyclophilin D–dependent oxidative stress.^28^ Similar benefits in terms of engraftment were not observed in the initial studies using CB-derived HSPC,^11^ despite the capacity of CsA to decrease CB-CD34^+^ cell proliferation.^12^ This may be due to the fact that the cells were not isolated and cultured in the continuous presence of CsA, as performed by Mantel *et al*. The more quiescent BM-derived CD34^+^ cells could be less sensitive to environmental oxidative stress, reflecting the benefits of CsA upon shorter exposure only during the *ex vivo* transduction process. Nevertheless, additional mechanisms could also be involved in this shorter exposure setting, no clear impact of CsA on the ROS levels in BM-derived HSPC could be detected. Moreover, CsH also seems to confer some degree of engraftment benefit to untransduced mPB-derived HSPC *in vivo* without affecting their proliferation *ex vivo.*^12^ Prior engraftment experiments with CB-derived HSPC were performed with lab-grade LV^11^ that could potentially induce some degree of bystander toxicity due to impurities in the vector preparation, thereby masking some potential benefit on engraftment. This may also be the reason why no benefit in engraftment was observed for mice transplanted with BM-derived CD34^+^ exposed to lab-grade vector to assess effects of CsA on the brain-repopulating progenitors.

Cultured HSPC progressively lose engraftment potential by recruitment into cell cycle and loss of adhesion molecules, thus impeding their homing into the niche and driving lineage commitment and differentiation.^6–8^ Indeed, all the one-hit conditions tended to engraft better compared to the standard two-hit protocol in agreement with previous reports that shorter culture of HSPC will result in higher engraftment levels into immunocompromised mice.^9,12^ Nevertheless, in the context of gene therapy, shorter protocols would not allow clinically relevant levels of gene marking to be reached in the absence of transduction enhancers, such as Rapa and CsA described here. Furthermore, it has recently been shown that the high vector doses currently required for clinically efficacious gene transfer directly impact *ex vivo* survival of HSPC and their *in vivo* repopulation capacity due to vector-mediated triggering of the p53 signaling cascade.^10^ Interestingly, CsA seems to mitigate this effect, despite increasing gene transfer efficiency into HSPC, as the benefit in engraftment of CsA-exposed cells was particularly evident early after transplantation, and was maintained in the BM of mice long term. Although the mechanisms behind these effects warrant further investigation, the benefit tjat the shorter CsA-based transduction protocol confers to early engrafting cells is potentially of clinical relevance, as rapid engraftment of short-term HSC is critical for a safe and successful clinical outcome. Overall, these findings contribute to the development of more efficient and sustainable LV gene therapy protocols, underscoring the benefits of scaling down required vector doses, as well as shortening the HSPC *ex vivo* culture time.

## Supplementary Material

Supplemental data

Supplemental data

Supplemental data
