# Supplementary material for: Assessing the Impact of Cyclosporin A on Lentiviral Transduction and Preservation of Human Hematopoietic Stem Cells in Clinically Relevant Ex Vivo Gene Therapy Settings
Source: Hum Gene Ther. 2019 Sep 17;30(9):1133–46. doi: 10.1089/hum.2019.016 (PMC6761585; doi:10.1089/hum.2019.016)
Supplement: Supplemental data [file Supp_FigS2-TableS1.pdf]

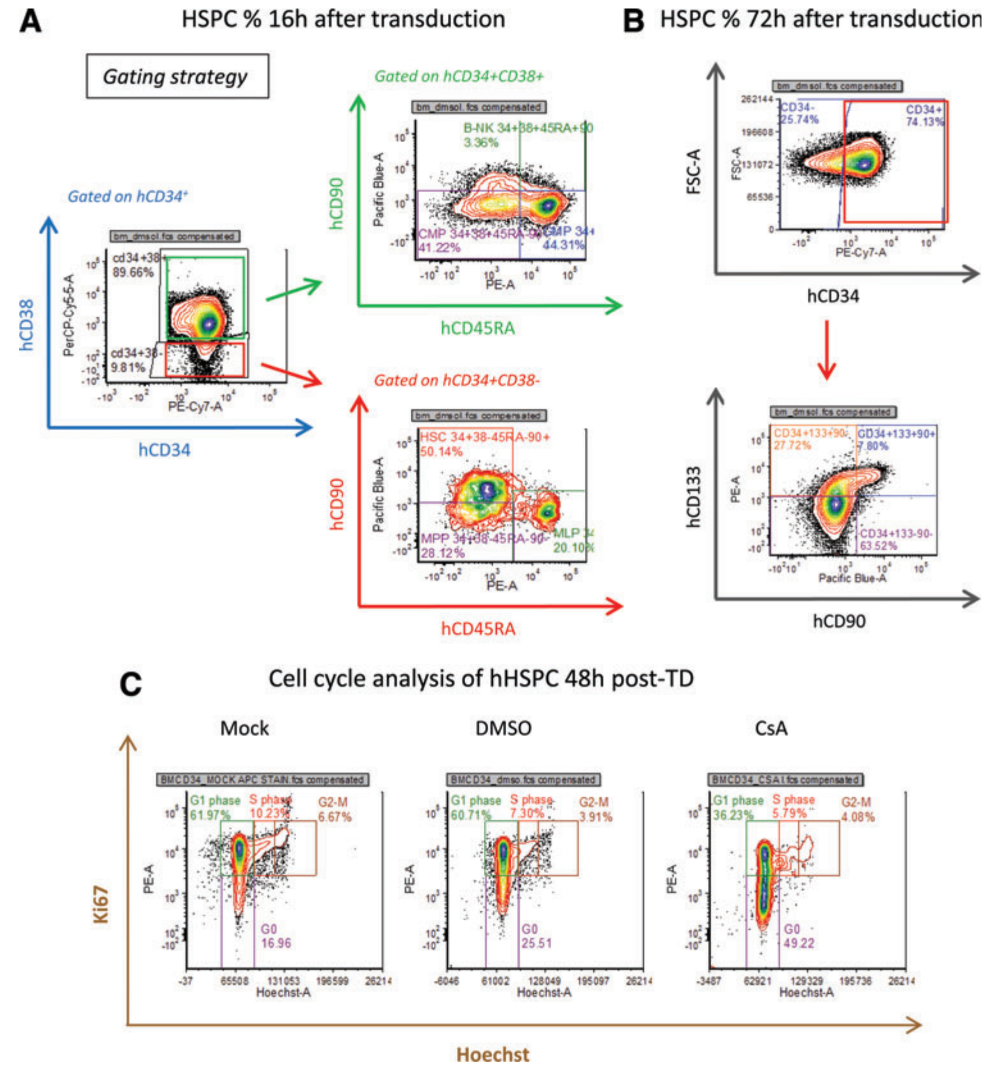

Supplementary Figure S2. The subpopulation composition of BM-derived CD34$^+$ HSPC was measured at **(A)** 16 and **(B)** 72 h post transduction using the reported gating strategies. **(C)** Gating strategy to measure the cell cycle in BM-derived CD34$^+$ HSPC 48 h post transduction.

Supplementary Table S1. *PCR reactions for ddPCR*

Cycling step	*Temperature (°C)*	*Time*	*Number of cycles*	
Enzyme activation	95	5 min	1	ddPCR 200
Denaturation	95	30 s	40	or 300 nM
Annealing/extension	63	1 min	40	
Signal stabilization	4	5 min	1	
	90	5 min	1	
Hold (optional)	4	Infinite	1	
