# Supplementary material for: Assessing the Impact of Cyclosporin A on Lentiviral Transduction and Preservation of Human Hematopoietic Stem Cells in Clinically Relevant Ex Vivo Gene Therapy Settings
Source: Hum Gene Ther. 2019 Sep 17;30(9):1133–46. doi: 10.1089/hum.2019.016 (PMC6761585; doi:10.1089/hum.2019.016)
Supplement: Supplemental data [file Supp_TableS2-S3.pdf]

Supplementary Table S2. *List of human antibodies used for flow cytometry*

Antibody	*Fluorochrome*	*Dilution*	*Clone*	*Company*	*Code*
hCD33	BV421	1:25	WM53	BD Biosciences	562854
Anti-human FCR blocking		1:50		Miltenyi Biotec	120-000-442
Mouse Fc block		1:100	2.4G2	BD Pharmigen	553142
hCD45	APCh7	1:25	2D1	BD Biosciences	641417
hCD19	PE	1:25	SJ25C1	BD Biosciences	345789
hCD33	PeCy7	1:25	P67.6	BD Biosciences	333952
hCD3	APC	1:25	UCHT1	BD Biosciences	555335
hCD13	BV	1:25	WM15	BD Biosciences	562596
hCD34	PeCy7	1:25	8G12	BD Biosciences	348811
hCD38	V450	1:25	HB7	BD Biosciences	646851
hCD90	APC	1:25	5E10	BD Biosciences	559869
hCD133/2	PE	1:25	293C3	Miltenyi Biotec	130-090-853
hCD45RA	PE	1:25		Miltenyi Biotec	130-092-248
hKi67	PE	1:10	B56	BD	51-36525X
IgG1 isotype control	PE	1:10	MOPC-21	BD	51-35405X

Supplementary Table S3. *Summary of samples used for integration site analysis and number of integration sites retrieved for each sample*

Vector	*Treatment*	*MOI*	In vivo *or* in vitro	*Sample ID*	*ng DNA analyzed*	*Number of IS*
SINLV-*GFP*	CsA	10	*In vitro*	CSA-CD34-CP-03	500	1446
		10	*In vivo*	CSA-CP-07	167	112
				CSA-DC-04	167	267
				CSA-DC-05	167	78
				CSA-DC-06	333	172
IDUA-LV		100	*In vivo*	CSA-CP-19-I	333	13
				CSA-CP20-I	500	29
SINLV-*GFP*	DMSO	10	*In vitro*	DMSO-CD34-CP-01	167	291
		10	*In vivo*	D-CP-04	167	33
				D-DC-01	167	121
				D-DC-02	333	90
				D-DC-03	333	82
IDUA-LV		100	*In vivo*	D-CP17-I	100	18
				D-CP18-I	100	10

in vitro, integration sites were retrieved from *in vitro* cultured HSPC after transduction with the indicated vector (2 weeks in culture); *in vivo*, integration sites were retrieved from BM cells from transplanted mice at 17 weeks after transplant; ng DNA analyzed, PCR reactions were performed on variable amounts of DNA, depending on availability.
