# Supplementary material for: Assessing the Impact of Cyclosporin A on Lentiviral Transduction and Preservation of Human Hematopoietic Stem Cells in Clinically Relevant Ex Vivo Gene Therapy Settings
Source: Hum Gene Ther. 2019 Sep 17;30(9):1133–46. doi: 10.1089/hum.2019.016 (PMC6761585; doi:10.1089/hum.2019.016)
Supplement: Supplemental data [file Supp_FigS1.pdf]

Supplementary Data

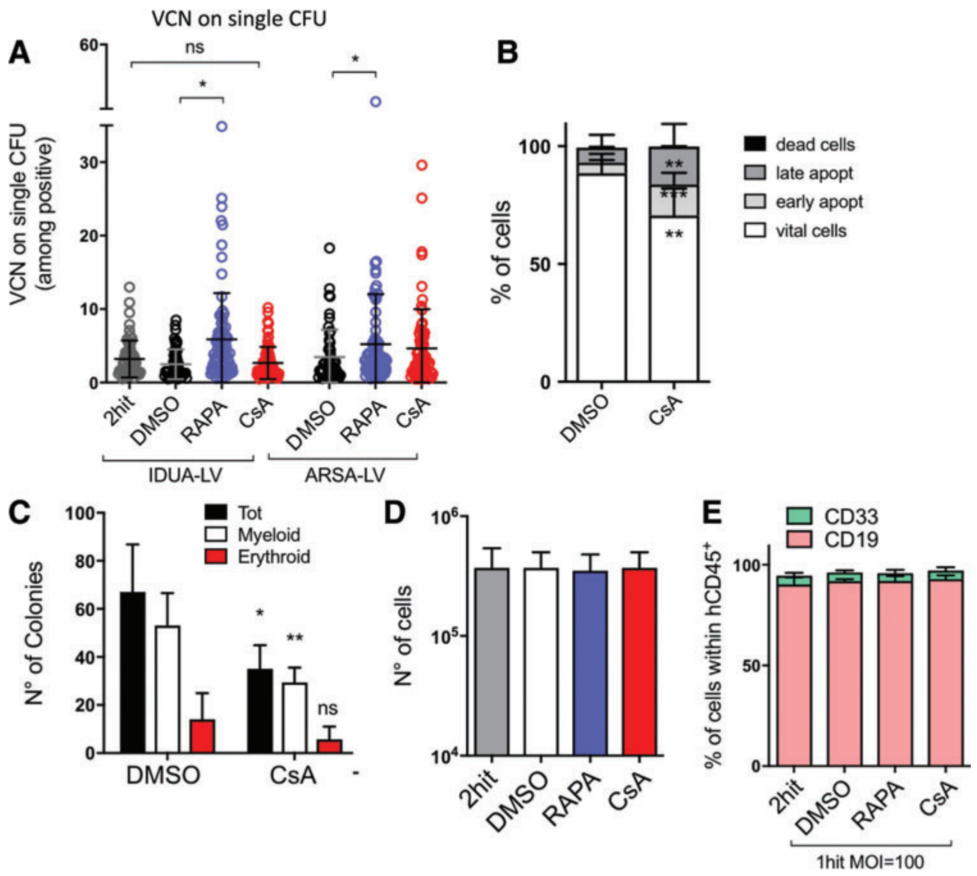

Supplementary Figure S1. **(A)** BM-derived CD34+ cells were transduced with *IDUA*-LV, as reported in Fig. 1A. VCN/human genome were evaluated, picking single colonies after 15 days in culture ($M \pm SD$, $n \geq 50$, Kruskal–Wallis with Dunn's multiple comparison test vs. each DMSO; ns, not significant; $*p \leq 0.05$). **(B** and **C)** mPB-CD34+ cells were transduced or not with a lab-grade PGK-*GFP* LV in the presence of DMSO/CsA, and apoptosis **(B)** or colonies count **(C)** were reported ($M \pm SD$, $n = 10$, Mann–Whitney test vs. each DMSO, $**p \leq 0.01$, $***p \leq 0.001$). **(D)** BM-CD34+ cells were counted at the time of transplantation in NSG mice, as reported in Fig. 1 for *IDUA*-LV. **(E)** Percentages of human B, T, and myeloid lineages within human cells were shown in the BM of mice from the experiments reported in Fig. 1 at 22 weeks post transplant.
